# Machine Learning Approaches for Myocardial Motion and Deformation Analysis

**DOI:** 10.3389/fcvm.2019.00190

**Published:** 2020-01-09

**Authors:** Nicolas Duchateau, Andrew P. King, Mathieu De Craene

**Affiliations:** ^1^CREATIS, CNRS UMR 5220, INSERM U1206, Université, Lyon, France; ^2^School of Biomedical Engineering and Imaging Sciences, King's College London, London, United Kingdom; ^3^Philips Research Paris, Suresnes, France

**Keywords:** machine learning, computer-aided diagnosis, myocardial motion, myocardial strain, cardiac imaging

## Abstract

Information about myocardial motion and deformation is key to differentiate normal and abnormal conditions. With the advent of approaches relying on data rather than pre-conceived models, machine learning could either improve the robustness of motion quantification or reveal patterns of motion and deformation (rather than single parameters) that differentiate pathologies. We review machine learning strategies for extracting motion-related descriptors and analyzing such features among populations, keeping in mind constraints specific to the cardiac application.

## 1. Introduction

### 1.1. Myocardial Motion and Deformation Analysis: What For?

Pump efficiency can discriminate failing from healthy hearts, as quantified by volume and ejection fraction. Clinicians are well aware of the limitations of these simple measurements to face the complexity of heart disease, and recommend finer markers of cardiac mechanical dysfunction (1). Myocardial motion (displacement or velocity) and deformation (strain or strain rate) are richer descriptors of (ab)normal cardiac function (2, 3). They can provide characteristic spatiotemporal signatures for disease at each location of the myocardium and each instant of the cardiac cycle. They are often projected onto anatomically-relevant directions to facilitate interpretations (4). Interestingly, they can be estimated from routine modalities such as echocardiography and magnetic resonance (MR) (5), and have therefore been thoroughly investigated for a wide range of applications.

### 1.2. Machine Learning for Myocardial Motion and Deformation Analysis: What For?

Machine learning builds upon models whose optimal parameters are learnt from a set of samples representative of the studied population. This data-driven approach is more flexible than traditional methods (e.g., variational), as demonstrated for myocardial segmentation (6, 7), and has strong potential for the analysis of complex descriptors such as myocardial motion and deformation. In essence, machine learning seeks to learn data representations (either explicit or hidden) for better solving a supervised problem or for characterizing the data distribution. This often involves dimensionality reduction to facilitate the analysis of high-dimensional descriptors, and requires navigating between the low-dimensional/latent space and high-dimensional/original space for better interpretation.

### 1.3. Which Data Approach for Learning?

**Figure 1:**
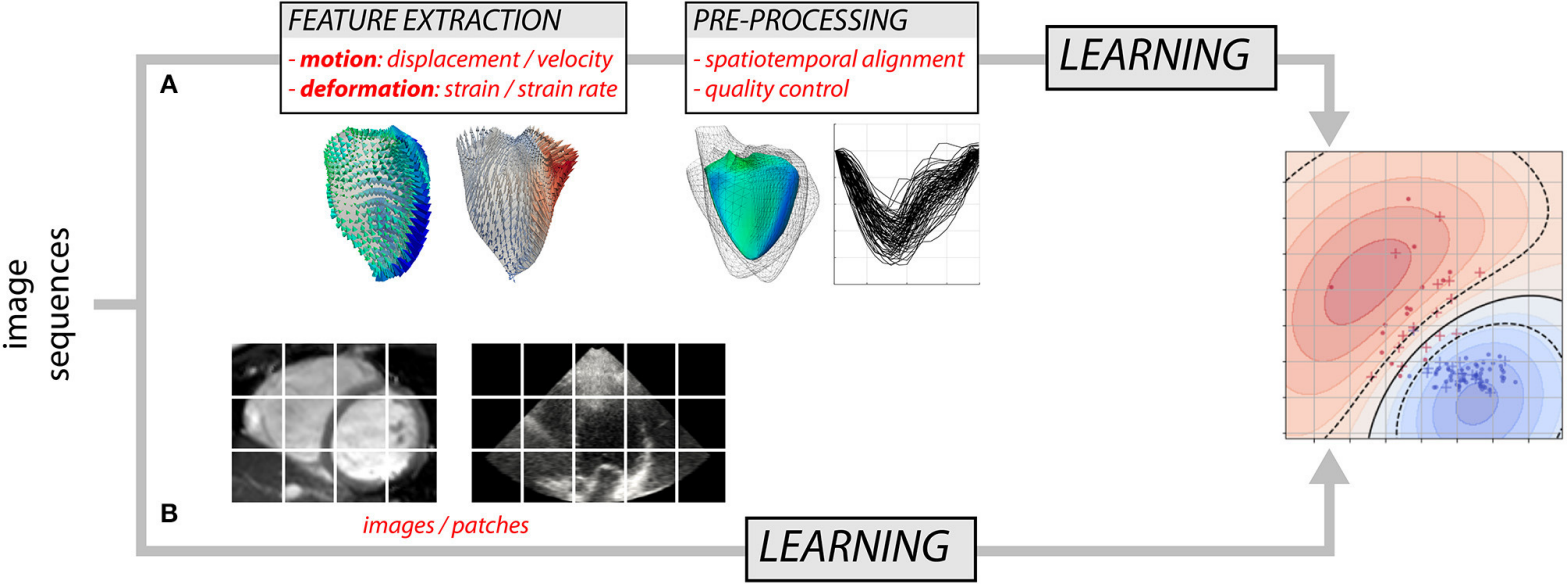
Two possible approaches for analyzing myocardial motion and deformation from image sequences using machine learning: **(A)** extraction of descriptors followed by their analysis, and **(B)** both parts addressed at once.

Over the years, researchers have gained detailed knowledge of the complexity of cardiac mechanics, and proposed physiologically-relevant motion and deformation descriptors, from global strain in a single anatomical direction to richer representations such as 3D+t vector or tensor fields. Most approaches decompose the analysis into two steps (Figure 1A): the extraction of motion/deformation descriptors from image sequences, followed by their analysis over a population of interest. Machine learning can address both parts, and we discuss these topics separately (sections 2 and 3). Deep neural networks (8) may address the two parts in Figure 1A, but also enable the analysis of population data directly from the image sequences by looking for image features not necessarily interpretable or visualizable, but optimal to answer the clinical question of interest (Figure 1B). We specifically comment on this strategy, which is more recent and preliminary, in section 4.7.

## 2. Motion and Deformation Estimation

Traditionally, myocardial motion fields have been estimated from images using standard image registration techniques such as optical flow (9), free-form deformation (10), or block matching (11). Naturally, this depends on the algorithm ability to catch motion-related structures, which strongly varies with the imaging modality. Tags and speckles can directly be tracked within the myocardium in tagged MR and 2D/3D echography (within the limits of tag fading, speckles temporal consistency, and out-of-plane motion), contrary to cine MR where algorithms tend to approximate motion from endocardial/epicardial contour tracking. A dedicated review (5) details the standards for spatial and temporal resolution and the influence of imaging parameters on the estimation of myocardial deformation.

Approaches based on neural networks challenge the variational formulation of motion estimation, as shown on video image sequences with the FlowNet2 convolutional neural network (CNN) architecture (12) that focuses on optical flow. Similar approaches have been applied to cardiac imaging (13, 14), but raise several methodological questions. First, the generalization ability of the trained networks to estimate a wide range of deformations *at multiple scales* still needs to be verified. This is critical for specific disease traits of lower prevalence. Furthermore, robustness to a variety of routine clinical imaging conditions (different image qualities, fields of view, devices, etc.) needs to be established. Second, supervised CNN-based motion estimators such as FlowNet2 do not embed any regularization, and are therefore sensitive to imaging noise if it differs from the training database. This not the case for unsupervised approaches like (13), which use an intensity-based loss, combined with a regularization term as in classical image registration. Finally, motion features can boost segmentation performances (15–17), as looking at several frames improves the manual segmentation of physicians. Further details are given in a review dedicated to deep learning for motion estimation in medical imaging (18).

Statistical models learnt from data can act as regularizers for tracking algorithms. (19) used dictionary learning as a sparse basis for cardiac motion fields to feed the regularization. Within deep learning, auto-encoders can encode spatial transformations into a low-dimensional space and provide powerful projection and reconstruction operators to connect with the tracking in the original image space (20).

Additional constraints specific to the cardiac application can provide more plausible registration outputs, such as invertibility (the myocardium does not fold) and incompressibility, as investigated for the diffeomorphic LogDemons (21) and free-form-deformation algorithms (22). Temporal consistency has been enforced through 4D representations of motion (23, 24), for multiple pairwise transformations simultaneously (25), or for intra/inter-subject mappings (26). Motion and deformation estimation with machine learning should also consider these aspects for better consistency and robustness.

## 3. Motion and Deformation Analysis

### 3.1. Before the Analysis: Data Normalization

Cardiac image data often need to be normalized in terms of anatomy, frame rate or cycle phases, before any statistical or machine learning analysis.

Image sequences can be registered using a 4D transformation model based on e.g., free-form deformation (10) or demons (26). This approach quantifies the spatiotemporal differences between the image sequences, analyzed statistically afterwards through deformation-based morphometry methods.

Motion or deformation descriptors (or any other data) from a given individual can also be transported to a reference template (generally, a central case at end-diastole). This involves local reorientation of the motion/deformation fields (27, 28), adjusted to the addressed clinical question (29). Temporal differences between sequences can also be normalized by resampling before the motion extraction [e.g., piece-wise linear interpolation (30)]. Recent approaches transport the whole subject-specific trajectory instead of the descriptors of interest, with specific computational considerations (31, 32). Automatically estimating multiple templates across the sequence may also be well adapted to the cardiac circular/periodic dynamics (33).

In both strategies, existing data correspondences facilitate the normalization. Spatial alignment can rely on anatomical landmarks (apex, valve ring, etc.) or point-to-point correspondences obtained from model-based tracking of the anatomy. Temporal alignment can use physiologically-relevant instants, such as the maximum contraction (10) or QRS and valve events (28).

### 3.2. Learning From Motion and Deformation Data

Machine learning can benefit a wide range of clinical problems. Unsupervised approaches learn a data representation that uncovers useful insights into the data distribution, but without explicit reference to a particular clinical question. Clustering and dimensionality reduction techniques fall into this category. Supervised approaches train a model for a specific task, and labels/annotations are provided as supervision. For example, diagnosing disease may involve binary labels for supervision (disease/healthy) and the task would be to predict these labels from the motion data. The type of labels determines the task addressed by the model: categorical labels mean classification, whereas discrete or continuous labels imply regression. Supervised approaches also involve learning a (lower dimension) representation of the data that facilitates the classification/regression, but this representation can be formed in an unsupervised or supervised way, as described below.

#### 3.2.1. Unsupervised Learning

Unsupervised motion and deformation analysis shares objectives with statistical atlases, regarding how to characterize variability across a population. Pioneering works directly applied a principal component analysis (PCA) on myocardial displacements at each spatiotemporal location (34) over a healthy population, later extended through the estimation of local abnormalities in the myocardial velocities of a given subject compared to a reference population (28, 35). However, these analyses consider each spatial location and temporal instant independently from the others. The statistical analysis can also consider the motion patterns over the entire cardiac cycle as high-dimensional objects, as simply demonstrated through a PCA on temporal strain traces concatenated over the heart segments (36, 37). This approach reminds earlier work on Active Appearance Motion Models (38), which statistically analyzed both displacement and image intensity information over the entire cardiac cycle.

More advanced strategies estimate a low-dimensional space that encodes the high-dimensional myocardial motion/deformation data and navigate through this space, although this requires specific care. Myocardial shapes across a population can be considered as originating from one or several references under the action of a transformation such as a diffeomorphic warping. In this case, the space of myocardial shapes is related to the (known) non-linear high-dimensional space of diffeomorphic transformations. This space is a manifold, and known tools exist to perform statistics on such transformations and therefore on myocardial shapes while preserving this data structure (39, 40). Myocardial motion/deformation patterns may also be considered as originating from a non-linear high-dimensional manifold, but in this case the manifold is unknown. Machine learning allows estimating this space from data, and can overcome the limitations of linear techniques such as PCA that ignore this known structure. A general framework (41) groups the vast variety of existing manifold learning techniques. A graph is built across high-dimensional samples to approximate the manifold, and diagonalization, and dimensionality reduction processes provide a low-dimensional space that encodes the data. Techniques generally differ on how input samples are related within the graph, either locally (e.g., distance between neighbors, or local structure variations expressed in the graph Laplacian) or globally (e.g., geodesic distance). These techniques improve the statistical analysis of myocardial motion and deformation patterns. They can represent the continuum of disease from normality while preserving the data structure (42). The unsupervised representation of populations is particularly interesting when existing labels are not fully trusted, as in heart failure with preserved ejection fraction (43, 44) or when a supervised formulation of the clinical problem is uncertain, such as outcome from cardiac resynchronization therapy (45).

Nonetheless, these techniques normally lack explicit mappings between the high-dimensional and low-dimensional spaces, which are typically approximated using out-of-sample reconstruction/regression (46) and are therefore inexact. Deep learning auto-encoders explicitly address this by simultaneously learning how to encode and decode high-dimensional data with a limited number of parameters while minimizing the reconstruction error. However, this also requires constraining the distribution of samples in the latent space so that a statistical analysis can still be performed on it afterwards, as in variational auto-encoders (47). These techniques are promising for the analysis of myocardial motion and deformation and start being used in cardiac imaging for segmentation (48, 49) or segmentation-based biomarkers (50).

#### 3.2.2. Supervised Learning

As noted above, designing a supervised learning model traditionally consists of two steps (Figure 1A). First, the input data are transformed to a new representation that facilitates the task performance. Second, a classification or regression model is trained to predict the label given the new representation. More recent techniques such as deep learning combine these two steps: the representation is learnt and optimized during the model training (Figure 1B). Below, we first summarize works using supervised learning in the traditional way and then we briefly review more recent deep learning approaches.

The new data representation can be estimated using knowledge of the labels (supervised way) or without such knowledge (unsupervised). In other words, although the final classification or regression model is supervised, the transformation to a new representation can be unsupervised. Examples include the dimensionality reduction methods reviewed in section 3.2.1, such as PCA (51–53) or non-linear manifold learning (53, 54). The use of hand-crafted features such as volumes/diameters/strains (55) and radius/thickness (56, 57) also falls into this category, although one could argue that knowledge of the task was also used to design these features. A supervised approach was taken in Dawes et al. (58), in which supervised PCA was used to find the principal components of displacement data related to survival.

Classification or regression come once the new representation is obtained. Many classification algorithms have been used, including support vector machines (SVM) (55, 59), random forests (55), variants of dictionary learning (59–61) and ridge logistic regression (57). Regression applications rely on svm (62) and multiscale kernel regression (54).

Recent research has increasingly focused on deep learning for both classification and regression from dynamic imaging data. In these approaches, the activations of intermediate network layers can stand as a transformed representation formed in a supervised way. Inputs to these models are commonly dynamic image intensity data, but segmentation data has also been used (63). For classification, variants of auto-encoders have been a common architecture choice. An auto-encoder is a deep learning-based dimensionality reduction technique, and classification can be performed in the low-dimensional latent space learnt without supervision (53), or in a supervised way by including classification accuracy into the loss function (48, 63, 64). Auto-encoders are attractive as they allow examining the classification features in the original image space, leading to more interpretable analyses. CNNs have also been proposed for classification (65), and a challenge on automated diagnosis was recently organized (7). Regression tasks such as estimating volume and/or ejection fraction may also involve CNNs (66), as tested on the recent Kaggle Challenge data^1^. Variational auto-encoders have also been used to perform regression in the latent space (50).

A wide set of classification applications involved myocardial motion or deformation, including identifying abnormal wall motion (59, 61), predicting therapy response (67) and survival (58, 64), and diagnosing myocardial infarct (16, 60, 65, 68) or pathology (7, 48, 57, 63). Regression applications aimed at localizing myocardial infarct (54), grading myocardial motion defects (62), and estimating volumes (66).

Detecting some form of abnormality is a common theme for supervised learning applications, for which two main strategies exist. In the first one, the transformed representation only involves healthy subjects: the distribution of samples in the low-dimensional space therefore represents healthy variations, and subsequent subjects who fall away from the healthy distribution are considered abnormal, as investigated on myocardial velocities (28, 35) and shapes (69). The other strategy learns a low-dimensional representation from both healthy and pathological subjects, where supervised classification can be applied afterwards (70).

## 4. Specificities of the Clinical Context

### 4.1. Physiological Consistency

Learning algorithms utilize a low-dimensional representation of the high-dimensional motion/deformation data, where the population variability is either rendered through diagonalization according to inter-subject distances, or correlated to labels of interest. Transforming to and from this representation involves interpolation between samples. Regularizing the low-dimensional space ensures smoother interpolation and generates new samples that are physiologically plausible (49, 71). In both of these works, the low-dimensional space produced by the encoding part of a CNN was regularized to map smoothly to a set of input shapes, labeled images, or slice locations. This notion of joint projection from the image and label space is also inherently present in more classical manifold learning techniques such as partial least squares. Similar notions need to be extended to motion fields, whilst mapping similar pathological conditions to close locations in the latent space.

### 4.2. Spatiotemporal Analysis

Most learning techniques consider high-dimensional inputs as high-dimensional column vectors or a set of patches, and disregard the spatiotemporal characteristics of motion and deformation. Few works explicitly addressed this issue for the statistical analysis of populations. A bilinear statistical model was used on cardiac shapes (72) to distinguish inter-subject variations from individual heart dynamics. (73, 74) explicitly addressed the problem through spatiotemporal tensor decomposition. Duchateau et al. (75) tuned up the contributions of the spatial, temporal, and magnitude dimensions to analyze changes in deformation patterns through registration. Jia et al. (31) and Guigui et al. (32) transported temporal trajectories without explicitly extracting motion or deformation descriptors beforehand. These strategies, limited to variability analyses, pave the ground for better considering spatiotemporal aspects with machine learning.

### 4.3. Interpretability

Many tasks may benefit from somehow “interpretable” learnt models, i.e., a user should have ways to inspect the input data characteristics that led to the output prediction or representation. The recent trend toward more complex learning models (such as deep learning) has raised the interest for this property, since these models are generally harder to interpret than simpler ones. One approach consists in defining a simpler model that is “locally similar” to the global complex model (i.e., it has similar performance for similar inputs) (76). For deep learning based approaches, “saliency maps” can be produced, which show which parts of the input data were important in producing the output. Alternatively, regression or autoencoders can be used to reconstruct cases from the low-dimensional latent space and examine features in the original-high dimensional space, with clear benefits for interpretability as demonstrated in Clough et al. (48), Puyol-Anton et al. (53), Biffi et al. (63), and Bello et al. (64).

### 4.4. Database Size and Heterogeneity

**Figure 2:**
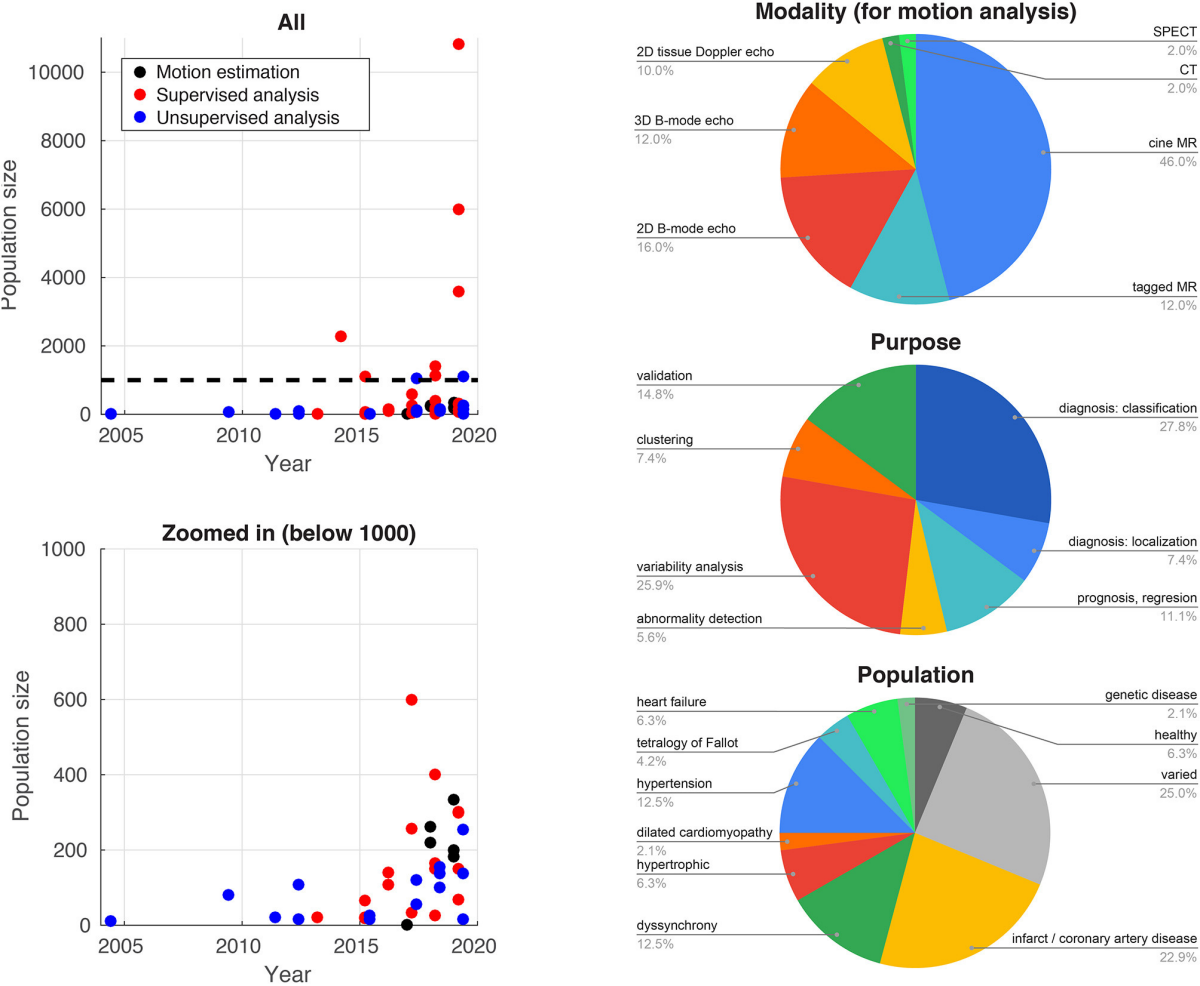
Database sizes (left) and distribution of imaging modalities, application purposes, and target populations for the studies cited in this paper that use machine learning for myocardial motion or deformation analysis.

Traditionally, difficulties in accessing and reliably annotating databases of medical images have led to smaller databases in medical imaging compared to computer vision applications. Recent initiatives such as the UK Biobank project^2^ (77) now provide large-scale annotated imaging databases, fuelling a rise in more data-intensive methods such as deep learning. Figure 2 illustrates this high increase over recent years for the studies reported in this paper. The impact of these large databases is high: reporting reference ranges for cardiac functional biomarkers is now possible with much greater confidence (78, 79), in addition to detecting effects otherwise hidden with smaller databases, as shown for genome data (77). Data heterogeneity is also crucial when choosing or curating a database for a specific task, i.e., the database should include sufficient subjects to cover a range of values for the output label and guarantee the model generalizability. More pathology-focused databases such as those in the Cardiac Atlas Project^3^ (80) have an important role to play in this respect.

### 4.5. Validation and Standardization Initiatives

As analyzing the tracking output is sensitive to processing errors, in particular for multi-centric data, tracking algorithms should be benchmarked to prevent bias due to different manufacturers or settings/practices. To ensure reproducibility of clinical decision-making from these data, standardization initiatives arose from academic, clinical, and industrial actors of cardiac imaging. Strain estimation was compared across vendors for synthetic and real images (81). Outputs were consistent regarding the differentiation between pathological and healthy regions, and the identification of ambiguous zones. However, statistically significant differences among vendors were reported, including differences around 15% for the biggest scars. These differences call for benchmarks on more realistic datasets (both regarding geometry and image quality), obtained e.g., from simulation frameworks that mix image formation and biomechanical models with real images (82).

Complementary standardization of imaging are also investigated through deep learning, for the control of e.g., the full coverage of the ventricles (83), the view/plane (84, 85), and the image quality in general (78) or due to motion-related artifacts (86).

### 4.6. Multiple Modalities/Descriptors

Most studies only consider a single type of motion or deformation descriptor at once from a single acquisition and a single modality, unlike clinical reasoning, which repeats acquisitions in the same or different modality and uses different types of measurements and descriptors. Recent works addressed these limitations within the framework of manifold learning. (30) enforced the complementarity of multimodal acquisitions (tagged MR and 3D echocardiography) using canonical correlation analysis and partial least squares methods. (87) used a similar strategy to better relate myocardial shape and deformation descriptors. Puyol-Anton et al. (70) investigated multi-view linear discriminant analysis for classification purposes. Finally, the more generic framework of multiple kernel learning allows reducing the dimensionality and examining the weights attributed to each descriptor. It was applied to supervised (67) and unsupervised (43–45, 88) problems, to investigate multiple descriptors among which motion-based ones, which could come from different modalities or different views of a single modality.

### 4.7. Complexity of the Models and Data Descriptors

Machine learning relies on models whose complexities should be adjusted to the question being answered. Researchers should keep in mind that such models only provide an approximation of reality, and try to minimize this error (e.g., by refining the model, adding more data or relevant descriptors, or estimating uncertainties). We strongly recommend to start with simple data descriptors and models, and carefully benchmark the retained methods against simpler models or even standard statistics.

Deep learning approaches allow circumventing the design of hand-crafted features (Figure 1B), and therefore go beyond a substantial limitation of standard machine learning. They mainly have been used for supervised problems and avoiding segmentation. The ACDC challenge (7) included a diagnosis challenge not necessarily requiring segmentation, although all participants opted for segmentation-based diagnosis. Regression-based estimation of cardiac parameters directly from images was proposed in (66, 89, 90), and may also strengthen the segmentation-based estimation of such parameters (91). However, as already pointed out, this direct strategy may also limit interpretability, and therefore transfer to clinical practice.

## 5. Conclusion

Machine learning offers wide possibilities to automate processing, and notably extract and analyze myocardial motion and deformation. Driven by advances in cardiac segmentation and large databases collection, there is potential for substantially improving the characterization of the cardiac function and impacting clinical practice. Changes cover the automation of time-consuming and user-dependent tasks such as feature extraction, higher performance on supervised problems such as (earlier) diagnosis, prognosis, and risk stratification, and new unsupervised data representations for knowledge discovery such as clustering or phenotyping. Nonetheless, motion and deformation are rich but complex high-dimensional data. Efforts need to be continued to reduce uncertainties, approximations, and crucial misinterpretations along the analysis pipeline, from careful problem definition, compliance with the mathematical and physiological data properties, algorithms benchmarking/validation/testing, and health actors' education.

## Author Contributions

All authors listed have made a substantial, direct and intellectual contribution to the work, and approved it for publication.

### Conflict of Interest

MD was employed by Philips Research Paris. ND and AK have research publications and/or projects with researchers and engineers from private companies, but this did not influence the contents of this review.

## Footnotes

^1^Available online at: https://www.kaggle.com/c/second-annual-data-science-bowl/data

^2^Available online at: https://www.ukbiobank.ac.uk/

^3^Available online at: https://www.cardiacatlas.org/
